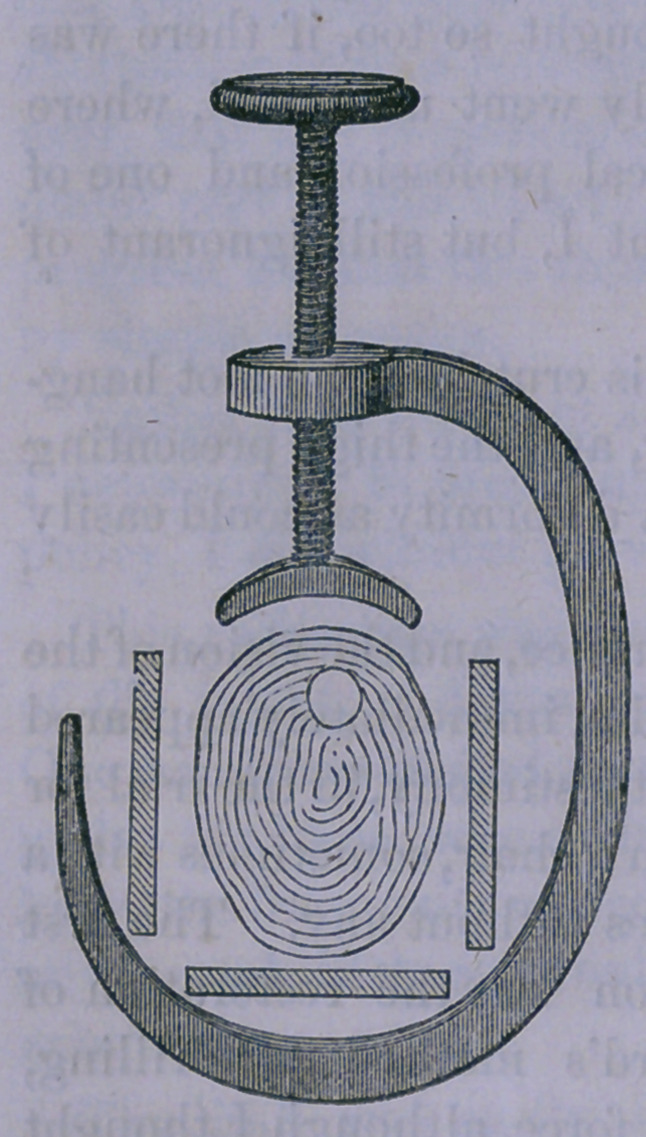# A Chapter of Accidents

**Published:** 1860-05

**Authors:** David Prince

**Affiliations:** Jacksonville, Ill.


THE CHICAGO

### MEDICAL JOURNAL.

VOL. III. MAY, 1860. NO. 5.

### Original Communications.

### ARTICLE 1.

### A CHAPTER OF ACCIDENTS.

AN OBLIQUE FRACTURE OF THE FEMUR—1½ INCH SHORTENING FROM PULLING OUT OF A LOOP OF ADHESIVE PLASTER—RUPTURE OF CALLUS AND RE-ADJUSTMENT AT THE END OF FIVE WEEKS—SPLINT KEPT ON SEVEN WEEKS FROM THE RE-RUPTURE—BONE BENT AFTERWARDS—BONE DRILLED FIVE OR SIX WEEKS FROM TIME OF BENDING, OR TWELVE OR THIRTEEN WEEKS FROM RE-RUPTURE—AFTER AN INTERVAL OF A WEEK, PRESSURE AND EXTENSION APPLIED, STRAIGHTENING THE BONE IN THREE DAYS—A LAW-SUIT FOR DAMAGES—NO VERDICT—REFERENCE TO AUTHORITIES ON SHORTENING—CASE OF DOUBLE FRACTURE—STARCH BANDAGE SHELL, AND A NEW WAY OF EXTENSION—REFERENCES TO AUTHORITIES ON BENDING OF BONE AND THE REMEDIES. BY DAVID PRINCE, M. D., Jacksonville, Ill.

James D. Reanch, a healthy boy 14 years of age, fell from a tree on the 3d of Octoher, 1859, receiving a simple oblique fracture of the upper portion of the middle third of the right femur. The direction of the fracture was from above, downward and outward, traversing a distance of three inches, measuring on the axis of the bone.

The limb was placed in Wilch's modification of Dessault's long splint. The extension was maintained by adhesive plaster, several strips, each about an inch wide, making a loop on one side—a similar appliance upon the other side of the leg—the plaster extending from the knee two-thirds of the way to the malleola, and a cord passing from each of these loops, and fastening below the foot-piece of the apparatus. The counter-extension was maintained in the ordinary way, by means of a perineal band of muslin enclosing cotton, and fastening (each end of it) to the upper end of the shaft of the splint.

All things went on favorably for four weeks, when one of the loops of adhesive plaster pulled out, unnoticed by the attendant, thus removing all tension and allowing the bone to shorten an inch and a half.

This event surprised me, because I had supposed that, after that period, the consolidation, in a healthy boy, would prevent shortening from a relaxation of the extension. It seems how ever, that the consolidation was slow, as shown in the subsequent part of this history, but not so far behind the ordinary progress, but that this shortening was too great to be remedied by simple extension.

Rather than turn out a case one inch and a half too short, I determined to break up the callus; which, for reasons of policy, I did without previous notice to the family.

This re-fracture was five weeks and two days from the date of the injury, and was accomplished with the greatest ease. One hand was placed upon the seat of fracture, and the other under the knee, when with but little force applied under the knee, the fragments ceased their hold upon each other without any sound such as would come from breaking solid bony substance.

The limb was easily extended to its proper length, and kept so satisfactorily for seven weeks more, when the callous seemed to be very firm. It was considered safe to take off the splint and give the boy more freedom of motion.

On careful measurement from the superior anterior spinous process of the ilium to the internal malleolus, and again from the trochanter major to the upper extremity of the fibula, the limb was found to be from a quarter to half an inch shorter than the other.

I gave the mother precautions not to allow sitting on a chair unless the foot was, at the same time, in another chair, the knee being stiff from confinement and involving of the muscles in callus, and that if the bone should be found to bend, to have the splint immediately re-applied.

I called as a matter of courtesy and precaution the next day, and found him dressed and sitting on a chair with his foot resting on a stool. He would not permit me to examine the limb, but my eye detected no deformity.

I dismissed the case from my mind, as a trouble ended, entirely satisfied with what I supposed the final result.

Three weeks afterwards a professional friend told me there was to be a consultation of Doctors over this case, and he thought I ought to be there. I thought so too, if there was anything the matter, and accordingly went uninvited, where I met three members of the medical profession and one of the legal. Rather ominous, thought I, but still ignorant of the case; I awaited developments.

Presently the boy came in upon his crutches, the foot hanging about three inches from the floor, and the thigh presenting a curvature of 20 or 30 deg.; such a deformity as could easily be recognized across a street.

I was thunderstruck with the appearance, and the vision of the boy on the chair, as I had last seen him, immediately appeared to me. It appeared from subsequent testimony, in the trial for mal-practice, that he sat *frequently* on a chair, sometimes with a support under the foot and sometimes without any. The first suggestion I made to the consultation was the restoration of the form of the limb, by Brainard's method of drilling, and afterwards applying mechanical force, although I thought mechanical force alone might succeed; as in Norris' note to Ferguson's Practical Surgery, (Ferguson Practical Surgery, with Notes by Norris: Lea & Blanchard, 1845; Page 340,) it appears that Dupuytren has succeeded as late as the 120th day from the receipt of the injury.

This was seventy days from the date of re-fracture, and twenty-one days from the time of taking off the splint, and there seemed a very good chance for succeeding by mechanical means alone, without first softening the callus by drilling.

I was not permitted by the family to apply this, nor even to see it done; and in the hands of another gentleman it failed after an application during two days.

Several weeks now elapsed without treatment, after which three perforations were made through the region of fracture, by Brainard's drill, Dr. English, assisted by Dr. Edgar, having the treatment of the case. After waiting a week, extension was applied by means of a long splint, to the distal end of which the mechanical power of Jarvis' Adjuster had been attached. Lateral pressure was made by a sort of tourniquet with a hook passing under a splint upon the posterior of the limb, and a screw pressing upon the convexity of the bone. It will be readily seen, that such an instrument would readily break down any bone, if the screw were forcibly turned.

The splint having been properly padded, the extension secured by adhesive straps, the counter-extension by a well cushioned perineal band fastening to the proximal end of the long splint, and the wooden ball of the screw of the tourniquet properly secured by gutta percha and padding, from pressing directly upon the prominent bony angle, extension was powerfully applied at the same time with latteral pressure, until a distinct yielding of the crooked bone was perceived. The lateral pressure was then slightly relaxed to avoid injurious pressure of the soft parts against the bone, but the extension was kept up unremittingly. In about three days the curvature disappeared, but as a precaution against accidental bending, the splint, with moderate extension, was kept on two weeks, and the thigh was afterwards protected by a starch bandage. Dr. English thinks there is from one-fourth to one-half inch shortening; Dr. Edgar thinks there is no shortening at all.

Here the surgical treatment ends, with an excellent result, notwithstanding two accidents, one of shortening and one of bending.

*Prosecution.*—In the February and March term of the Circuit Court of Morgan County: Justice D. M. Woodson presiding; J. L. McConnel and H. P. McClure for the prosecution; D. A. & T. W. Smith and Cyrus Epler for the defence. Damages claimed, $5,000—the jury disagreeing.

The prosecution attempted to maintain—

First, That the shortening of the limb was prima facia evidence of unskillfulness.

Second, That the breaking over of the limb without apprising the family and obtaining their consent, and having a consultation of surgeons and obtaining their approval, was an outrage.

Third, That the thigh bone must have been in a crooked state when the splint was taken off, because it was proved, by two unprofessional witnesses, who saw the limb through the clothes, to have been crooked a few hours afterwards. Dr. English thought that if the bone had been of proper shape at the time of taking off the splint, it could not have bent in so short a time without great pain. There was no direct testimony on this point.

On the other hand, it was maintained by the defense—

First, That the shortening resulted from an accident.

Second, That the re-fracture was the proper remedy, and in view of the surgeon's responsibility he was justified in putting it out of the power of parents or adverse medical council, to hinder his restoring the limb to its proper length.

Third, That a curved condition of the limb, without shortening, was a mechanical impossibility; that a loose application of the apparatus which would allow curving would certainly have resulted in shortening; that the callus has very little if any sensibility and that the absence of pain during the curving is no evidence of the absence of the curving itself.

I have this moral to draw from this lesson.

I. Let the surgeon apprize the patient and friends of the difficulties of the case, and the uncertainty of a perfect result, whatever skill and attention may be exercised.

II. Let him instruct the patient and nurses in the working of the machinery, and direct them to give him immediate notice of anything giving way or seeming to go wrong.

III. Let him not only caution the patient to be careful of the limb after the chief support is taken off, but let him apply a starch bandage or other support to the limb, to be worn long after the patient goes about.

IV. Last, but not least, let him be sure that he has *disinterested witnesses* to what he may say and do, so that if any accident or unfavorable result occurs, he may be able to prove his points.

Had I been careful on this last point, I should, probably, not have been sued for mal-practice.

*References.*—I subjoin the following references for the advantage of any who may have the misfortune to require them, in justification of practice and its results, in treatment of fracture of the femur or in defence against ignorance, malice, and cupidity.

*Length of time a splint should be kept on.*—Dr. Charles A. Pope, Professor of Surgery in the St. Louis Medical College, in his deposition in this case, says: "Seven weeks would generally be sufficient time for wearing the splint, but the patient should not use the limb for several weeks more.

Miller's Principles of Surgery, Philadelphia edition, 1845, page 497, "At the end of the fourth or fifth week our substitutes (for provisional callus) may be discontinued."

Linton's Practical Surgery, with Notes by Norris; 1842; page 83—bottom: "At least six weeks."

Hamilton on Fractures, page 433: "Eight weeks, though it may be safe (to remove the splints, in some cases) in six weeks." * * * * "The extension may generally be relaxed as soon as the 28th day."

In my case an accidental relaxation just about this time, resulted in shortening.

*Shortening.*—Dr. Pope, in his deposition in this case, says: "It is impossible to prevent shortening in some cases of oblique fracture of the thigh bone, for besides the physiological primary or immediate contraction or shortening of the muscles, there is also a physiological subsequent shortening, caused by the effusion of plastic material between and among their fibres, whereby their extremities are approximated, just as water has the effect of shortening ropes which were previously dry."

"Malgaine broke the thigh bone of a rabbit, and a few ounces weight sufficed to overcome the first shortening. Ten days subsequently it required fourteen pounds to produce any elongation of the muscles, and then only after their complete rupture."

Dr. Pope thinks "the average shortening is from one-fourth to one-half inch."

In the admirable work on Fractures and Dislocations, by Dr. F. H. Hamilton, of Buffalo, recently published, will be found an almost complete list references to the recorded opinions of surgeons, with regard to the practicability of avoiding shortening and the results actually obtained. As this work is, or ought to be, in the hands of every man who is liable to be called upon to treat fractures, it is not necessary here to enumerate the references, for the list, commencing on the 398th page, is very long.

The opinion of Malgaine and Maclise, that union of oblique fracture of the thigh without shortening is impossible, is expressed in very strong language. Malgaine says "the thing is simply impossible."

Maclise says "Out of every six fractures of the clavicle or thigh bone, I believe that as the result of our treatment by the present forms of mechanical contrivances, there would not be found three cases of co-aptation of the broken ends of the bone, so complete as to do credit to the surgeon."

Hamilton (page 404) concludes "that in case of an oblique fracture of the shaft of the femur, occurring in an adult, whose muscles are not paralyzed, but which offer the ordinary resistance to extension and counter-extension, and where the ends of the bones have once been completely displaced, *no*

*means* have yet been devised by which an overlapping and consequent shortening can be prevented."

"When, in consequence of displacement, overlapping occurs, the average shortening in simple fractures, where the best appliciances and the utmost skill have been employed, is about three-quarters of an inch."

This corresponds very nearly with the statistics of the New York Hospital, published by Dr. Lute, and quoted by Hamilton, in his book, and also in his Report on Deformities after Fractures, in the 10th volume (1857) of Transactions of the American Medical Association.

In addition to the opinions of American surgeons, quoted in the work of Hamilton, the opinions of Drs. Trowbridge Barnes, Burwell, and Austin Flint; also, that of Dr. Willard Parker, of New York, are given in the Report on Deformities after Fractures, page 271.

Hamilton gives a sufficiently extensive list of those confident surgeons who claim to have treated oblique fractures of the thigh without any shortening or deformity whatever, and especially refers to Liston, who says, (Liston's Practical Surgery, by Norris, 1842, page 86,) "By adopting the straight position, the surgeon has it *fully* in his power to preserve the limb of its original length and proper contour."

Notwithstanding this confident language, any one of any experience in the treatment of these injuries must see at a glance that the use of the long splint, as figured in Liston's book, having no means of constant extension, except by drawing from time to time upon the counter-extending band, must result in some degree of shortening as the rule; and Hamilton (page 403) quotes from Gamgee's work on the starched apparatus, (London, 1853, pages 54 and 55,) to prove that in the practice followed by Liston himself, in the University College Hospital: "The patients admitted with broken thighs or legs, were frequently discharged with manifest deformity."

Before leaving this subject of shortening, I will say, with reference to the starched bandage splint, that I have used it with great satisfaction in connection with permanent extension by means of the ordinary long splint, but I should not dare to trust the starched splint alone where the fracture is oblique. In one instance I used a method of extension which may, perhaps, be new.

Having been called in the middle of the night, several miles into the country, to see a patient who had fallen from a tree, and received an oblique fracture of the femur in its lower third, in a direction from above downward and inward, terminating in the upper part of the internal candyle, and another fracture of the same bone, at the junction of the middle and upper thirds, and having no appliances with me whatever, I prepared a long splint with a wide foot-piece, so as to carry the splint well off from the limb, and by means of muslin roller bands upon the ankle, and the ordinary extending perineal band, cushioned with cotton, I made extension while the patient was under the influence of a mixture of ether and chloroform. While the patient was still asleep I enveloped the whole limb, from the ankle to the groin, in a firm starch bandage, using plenty of pasteboard in its construction. During all the time of application of this shell of muslin, pasteboard, and paste, the extension was kept very tight, so as to make the broken limb seem a little longer than the other. A few hours afterwards it was deemed advisable to split this shell on the outside, opposite the seat of the lower fracture, but it did not become too tight from swelling of the limb at any other point. In two or three days it required to be split down and bandaged tighter, to make it grasp the limb firmly. The patient complained of the tightness of the extension on the top of the tarsus and around the lower portion of the leg, and I applied a strong loop of several thicknesses of adhesive plaster on each side of the leg, and in addition to a few circular turns of adhesive plaster for security, I made it still more firm by an additional shell of starch bandage over the whole limb, including the adhesive plaster, except the loop ends, which were made to project through this outer shell.

My improvised splint was exchanged for Day's long splint with a screw extension at the foot. By means of a cord through each loop, and fastened over the foot piece of the apparatus, with a carefully arranged counter-extending band, well cushioned, and a piece of soft muslin, well besmeared with tallow, applied next the skin of the groin, and all made tight, the knee elevated a little at the time of the first dressing, to take off the otherwise painful tension from the flexors of the leg, and kept thus slightly elevated, my patient was in as safe and comfortable a position as could be possible. This extension upon the starch bandage shell applied to a very large surface, the top of the tarsus and the swell of the malleoli, and the swell of the gastrocnemii muscles. The pressure of the shell upon the malleoli and tarsus was never in the least painful. As the contour of the thigh lessened, the shell was again split down on the outside, and drawn closer. The fingers passed along through the slit and under the shell over the seats of the fractures, discovered no enlargement from callus, thus favoring the doctrine that callus is, in great part, occasioned by motion and mal-position.

The patient came out without any appreciable deformity or shortening, by my most careful measurement, from superior anterior spinous process of ilium to the internal malleolus. Two things are necessary to such a good result:

1st. An extension that never relaxes, except by the stretching of the parts drawn.

2d. Such a close bandaging of the muscles as will stop all motion, either voluntary or spasmodic.

Here I cannot avoid a criticism upon the excellent book on surgery, by Erichson. Such language as this I think exceedingly dangerous to those who have not sufficient experience not to be hurt by it.

"By employing the starch bandage, I scarcely ever find it necessary to keep patients in bed with simple fracture of the thigh, for more than six or seven, or of the leg, more than three or four days, thus saving much of the tediousness and danger of the treatment." (Blanchard & Lea's edition, 1859, page 195.)

Here follows a picture of a man on crutches, with his limb hanging in a starched bandage shell. The pressure of this apparatus upon the upper part of the thigh, the tuberosity of the ischium, and the groin, must cause absorption of subcutaneous fat, and shortening must be inevitable in oblique fracture.

*Bending of recently united fracture, and the means of removing the deformity.*—Miller's Principles of Surgery, page 497.

"A broken bone must be warily used for some considerable time after apparent consolidation, and a broken bone may, anywhere, have its contour remedied by suitable pressure, even after the process of reparation seems to have been completed. * * * * Many a fractured limb has been set free at the ordinary time, of proper length, and void of all deformity, which, nevertheless has soon become both shortened and bent to an extent which has impaired both its symmetry and its function."

Hamilton's Report (loco citato) pages 271 and 272.—A remarkable case of bending occurring six months after the fracture.

Fergusson's Practical Surgery, page 341, note by D. Norris. The means for removing deformities following fractures of these kinds, viz:

1. Well applied pressure and extension.
2. Re-fracturing the bone.
3. Removal of a triangular section of bone at the most projecting portion of the deformed bone.

Pressure and extension is applicable as late as the fiftieth or sixtieth day from the date of the injury.

Dupuytren has succeeded as late as the one hundred and twentieth day from the injury.

4. The fourth method, introduced by Dr. Brainard, of Chicago, consisting in inducing inflammation and consequent softening of the bone; after which pressure and extension are to be made, supercedes the necessity for re-fracture of the bone or the removal of a triangular portion.

A full account of this method, by drilling the bone, is given in Dr. Brainard's Prize Essay, in the Seventh Volume of the Transaction of the American Medical Association, for 1854, page 254, and also in the *Chicago Medical Journal* for January, 1859, and favorably referred to in Hamilton's work on Fractures.

When the surgeon finds that in consequence of an undetected relaxation of extension, the fragments have overlapped, and a forcible and continuous extension fails to bring them again to their proper relations, there is, of course, nothing left but to break up the callus, or leave the patient to his deformity. As the callus is, for a considerable time, softer than the original bone, it will yield rather than the fragments themselves, after which, by stretching all the muscles passing the fractured point, as much as some of the muscles are stretched in some dislocations, the fragments may be brought to their proper relations and retained there until consolidation again takes place.

## Figures and Tables

**Figure f1:**